# A Harmonized Dataset of High-Resolution Embodied Life Cycle Assessment Results for Buildings in North America

**DOI:** 10.1038/s41597-025-05216-0

**Published:** 2025-07-01

**Authors:** Brad Benke, Manuel Chafart, Yang Shen, Milad Ashtiani, Stephanie Carlisle, Kathrina Simonen

**Affiliations:** University of Washington, Seattle, USA

**Keywords:** Environmental impact, Environmental health, Engineering

## Abstract

Building design practitioners are increasingly using life cycle assessment (LCA) to assess the environmental impacts of their buildings. However, industry-generated LCA results are rarely compiled into comparable datasets and rarely made public. Thus, harmonized and open-access datasets of building LCA results are limited, particularly in North America. Here we present a novel high-resolution dataset of building design characteristics, life cycle inventories, and environmental impact assessment results for 292 building projects in the United States and Canada. The dataset contains harmonized and non-aggregated LCA model results across life cycle stages, building elements, and building materials to enable detailed analysis, comparisons, and data reuse. It includes over 90 building design and LCA features to assess distributions and trends of material use and environmental impacts. Uniquely, the data were crowd-sourced from designers conducting LCAs of real-world building projects. This dataset fills critical gaps for the building industry, research, and policy communities, enabling them to analyze and compare the impacts of buildings, test or set performance targets, and motivate sustainable design and construction practices.

## Background & Summary

In 2022, buildings accounted for approximately 37% of global energy and process-related carbon dioxide (CO_2_) emissions, including 27% from building operations and 10% from building materials and construction^1^. Global building construction is projected to grow substantially through the 21st century^2–4^. While global progress is being made to decarbonize building operations, equal efforts to decarbonize building construction and materials are still needed^5^. Additionally, building materials are responsible for other significant land, air, and water emissions, which result in further environmental pressures such as eutrophication, acidification, and smog formation^6^. These material impacts, collectively referred to as embodied impacts (EI), or embodied carbon (EC) when referring to their global warming potential (GWP) only, are the result of material extraction, manufacturing, transportation, construction, use, and the eventual disposal of materials at the end of a building’s serviceable lifespan as outlined in international life cycle assessment (LCA) standards^7–9^. Addressing and reducing these emissions is essential to mitigating climate change and alleviating associated environmental pressures.

In response, researchers have increasingly focused on analyzing the EIs of buildings to identify trends, establish benchmarks, and inform policy development for reducing the environmental impacts of materials, buildings, and cities^10–12^. At the same time, research on building material stocks (the types and amounts of materials used by buildings) has also been growing. This research is valuable for linking global resource use to environmental impacts, enabling material flow analyses and assessing global or regional resource demand^13–17^. Material use intensity (MUI), a common metric in this field, is a measurement of a building or material’s mass per building floor area (e.g., kg of material per square meter). But despite their importance, free, openly accessible, and robust datasets on building-related EIs and MUIs are limited. Existing available datasets primarily focus on aggregated summaries of EIs from building LCAs and literature reviews^18–21^ or on building MUIs derived from various sources^22–28^.

Further, most existing studies and datasets struggle with comparability due to methodological inconsistencies in underlying LCA modeling, data collection, and reporting. Broadly, we refer to these inconsistencies as “harmonization” issues among existing studies and datasets, which we view as a spectrum (more or less harmonized) rather than a binary. Ultimately, few datasets integrate EIs and MUIs, and fewer still provide access to high-resolution and harmonized LCA results that enable more detailed analysis and comparison-making. This represents a significant gap in the field.

### Environmental impact datasets

The results of building LCAs contain data on the midpoint indicators of potential environmental burdens of building projects. These data, often derived from the building LCA models of design practitioners, represent a rich and growing source of information with many potential applications for researching the environmental impacts of buildings and influencing city^29^, state^30–32^, and national policies^33–35^. Most commonly, these include assessments of embodied carbon intensity (ECI), a measurement of a building’s total GWP per floor area. However, a lack of consistently applied building LCA standards, guidelines, modeling methods, and background datasets often leads to disparate and incomparable ECIs across different geographies, industries, and individual LCA modelers^36,37^. Ultimately, these harmonization issues significantly limit the interpretation and broader application of LCA data to address environmental challenges.

Simonen *et al*.^18^, as part of the 2017 Embodied Carbon Benchmark Study^19^, published a dataset on the ECIs of over 1000 buildings from around the world. This dataset, collected from the LCAs of private companies, existing research, and publicly accessible datasets, marked a foundational step in our research and this field. It remains one of the only datasets with a significant sample of projects from North America (representing 637 buildings). However, as it contains non-harmonized LCA results generated using varying LCA scopes and methods, the comparability of the data and its applications are highly limited. Additionally, the EC results are reported in aggregate (as project totals only), without breakdowns by life cycle stages or building elements, further reducing their usability and interpretability for benchmarking or detailed analysis.

In contrast, Röck and Sorensen compiled a dataset^20^ of EC impact data for over 800 European buildings from various sources to support benchmarking in Europe^21^. Their efforts to harmonize LCA results included normalizing data to a consistent reference study period (50 years). While this dataset provides aggregated results by different life cycle stages and building elements, which greatly increases its value, it contains large numbers of missing data points due to diverse LCA methods used in the original studies, which introduces potential biases and limits the dataset’s comprehensiveness.

Despite their contributions, both datasets focus exclusively on EC, excluding other critical EIs. Additionally, both relied on data generated using misaligned LCA methods, creating challenges for comparative analyses or integrating findings across regions. Addressing these issues requires further efforts to standardize LCA methodologies and enhance data harmonization. These datasets could also improve by expanding their scope to include a wider range of EIs and reporting full and detailed LCA results rather than aggregated summaries to foster collaboration across industries and geographies for comparative analysis and benchmarking.

### Material use datasets

As there is a strong correlation between global resource use and environmental degradation^38,39^, datasets on the quantity and intensity of materials used in buildings are also valuable for tracking the environmental impacts of buildings. These datasets may include total material quantities used in buildings but more recently tend to focus on MUIs. Such datasets can enable other researchers to perform material flow analyses or pair the MUIs with other emerging research and data^40,41^ on the average emissions intensities of building products. These approaches can be used to model the environmental impacts of future construction growth and resolve many of the methodological differences of the environmental impact-only approach, where LCA impacts are compiled and aggregated from various (typically non-harmonized) sources.

Examples of material use datasets include Heeren *et al*.^22,23^, who collected a dataset of over 300 global (but predominantly European) building projects from existing literature, which included MUIs for concrete, steel, wood, and over 20 other common building materials. Other regional-scale datasets have also been developed. Yang *et al*.^24^ compiled data for over 800 buildings in China; Sprecher *et al*.^25^ analyzed more than 60 Dutch buildings; and Guven *et al*.^26^ examined over 70 buildings, primarily in Canada. Uniquely, Guven *et al*. compiled the material quantities using takeoffs from construction drawings and categorized the materials using UniFormat and MasterFormat CSI divisions, two widely adopted North American construction classification systems. More recently, Fishman *et al*.^27,28^ extended the work of Heeren *et al*.^22,23^ by compiling global ranges of MUIs for over 800 buildings but focused only on structural materials from existing literature.

While these datasets represent significant contributions to the field, several limitations persist. Few MUI datasets adhere to a consistent framework for categorizing building materials. Fewer still reflect the material classification systems utilized for functionally equivalent products as established in product category rules or environmental product declarations (EPDs), making reuse of the data for environmental analysis challenging. Thus, many such datasets are limited in their broader applications for material research and policymaking. Additionally, without the associated environmental impact intensities of the materials, MUI datasets alone may not be as immediately actionable for understanding and mitigating EIs from materials and buildings, which designers and policymakers must seek to do before buildings are constructed. There is also a notable scarcity of MUI datasets generated by industry design practitioners performing LCAs of real-world buildings. Lastly, most existing datasets primarily focus on the structural components of buildings, overlooking material data for non-structural components, which are critical for comprehensive environmental assessments.

### Combined approach datasets

We identified limited datasets that combine material-level data and EI impacts of building projects in openly accessible formats, though recent attempts have been made. Röck *et al*.^42,43^ produced a global dataset, compiled from several previously referenced datasets alongside other sources, encompassing aggregate whole life carbon assessment results and MUIs for over 1200 buildings. While this dataset begins to fill a critical research gap on the whole life carbon impacts of buildings, it contains only 30 building projects from North America and is limited to reporting on the top five materials per project. Further, many of the dataset’s features are incomplete which limits sample sizes when attempting high-resolution analysis and comparison making. Junclaus *et al*. analyzed and published a dataset^44,45^ of MUIs and ECIs based on the authors’ own LCAs of the US Department of Energy (DOE) residential prototype models. This dataset and its associated supplementary tables provide valuable insights into the EC and MUI of residential buildings following consistent LCA modeling methods. However, it is based solely on hypothetical energy code reference models that do not capture the variability and complexity of real building projects.

We were unable to identify any openly accessible databases that exclusively represent real building projects, integrate both material use and environmental impact intensities, and are specifically regional to North America. Given North America’s significant contributions to global GHG emissions (the US in particular^46^), this absence represents a major gap in open-access data on the environmental impacts of buildings and building materials for some of the world’s largest emitting countries. Additionally, we found limited datasets that provide environmental impact results for midpoint indicators other than climate change (GWP) such as acidification, eutrophication, smog formation, ozone depletion, or non-renewable energy demand. These indicators are common outputs of LCA model results, closely linked to critical planetary boundaries^47,48^, and are prescient considerations for research on sustainable design and construction. Moreover, the recent growth in research on absolute environmental sustainability assessments has shown the significant value of LCA data beyond GWP for more comprehensively assessing the environmental burdens of anthropogenic systems^49–51^. Lastly, we found limited datasets that compiled full LCA model results that were conducted using consistent and harmonized LCA scopes and modeling methods. Openly accessible datasets of EIs, MUIs, and full LCA model results remain highly desirable but are currently nearly non-existent.

### Comprehensive datasets

We believe a more comprehensive approach to publishing life cycle assessment data is needed. Such an approach would include highly detailed reporting of building design characteristics (to determine functional equivalence), non-aggregated life cycle inventory results (to assess material use), and non-aggregated life cycle impact assessment results (to assess the EIs of the buildings and their materials). This would necessitate a novel data structure that enables high-resolution analysis and comparison-making, even when datasets are incomplete. Additionally, a comprehensive approach would strive for increased data harmonization, targeting not only the syntaxes, structures, and semantics of the final dataset, but also the underlying methods used to generate, classify, and report LCA data and results.

In response, the Carbon Leadership Forum (CLF) Benchmark Study v2^52^ was initiated to collect and harmonize building LCA data and associated project information of real-world buildings across North America. Here we present a dataset from the study that encompasses highly detailed and harmonized building design characteristics, life cycle inventory results (total material quantities and MUIs), and life cycle impact assessment results (including six TRACI^53^ environmental impact categories and their intensities) for 292 building projects across the United States and Canada. In total, the dataset represents nearly 5 million square meters of newly constructed floor area. Data were sourced from aligned LCA model results conducted by 30 design companies across North America who voluntarily contributed data to the study. The dataset contains detailed material and environmental impacts harmonized across life cycle stages, building elements, and building materials, enabling robust comparisons and promoting data reuse. It features a novel and non-aggregated data structure that allows for high-resolution and flexible analysis across multiple scales and dataset features. By publishing full LCA results in this way, other researchers can use the dataset to make specific comparisons in ways that were not possible with the aggregated data summaries of existing datasets outlined above.

To our knowledge, this represents the largest and most comprehensive dataset of its kind currently available. It aims to enable designers, owners, researchers, and policymakers to analyze and compare the impacts of buildings, set performance targets, motivate impact reductions, and better identify trends in material use, environmental impacts, and building performance. We hope this catalyzes future initiatives within the field to expand the scope and scale of LCA data collection, encompass broader geographic regions, and achieve higher levels of detail and methodological harmonization to support research on sustainable design and construction practices.

## Methods

**Fig. 1:**
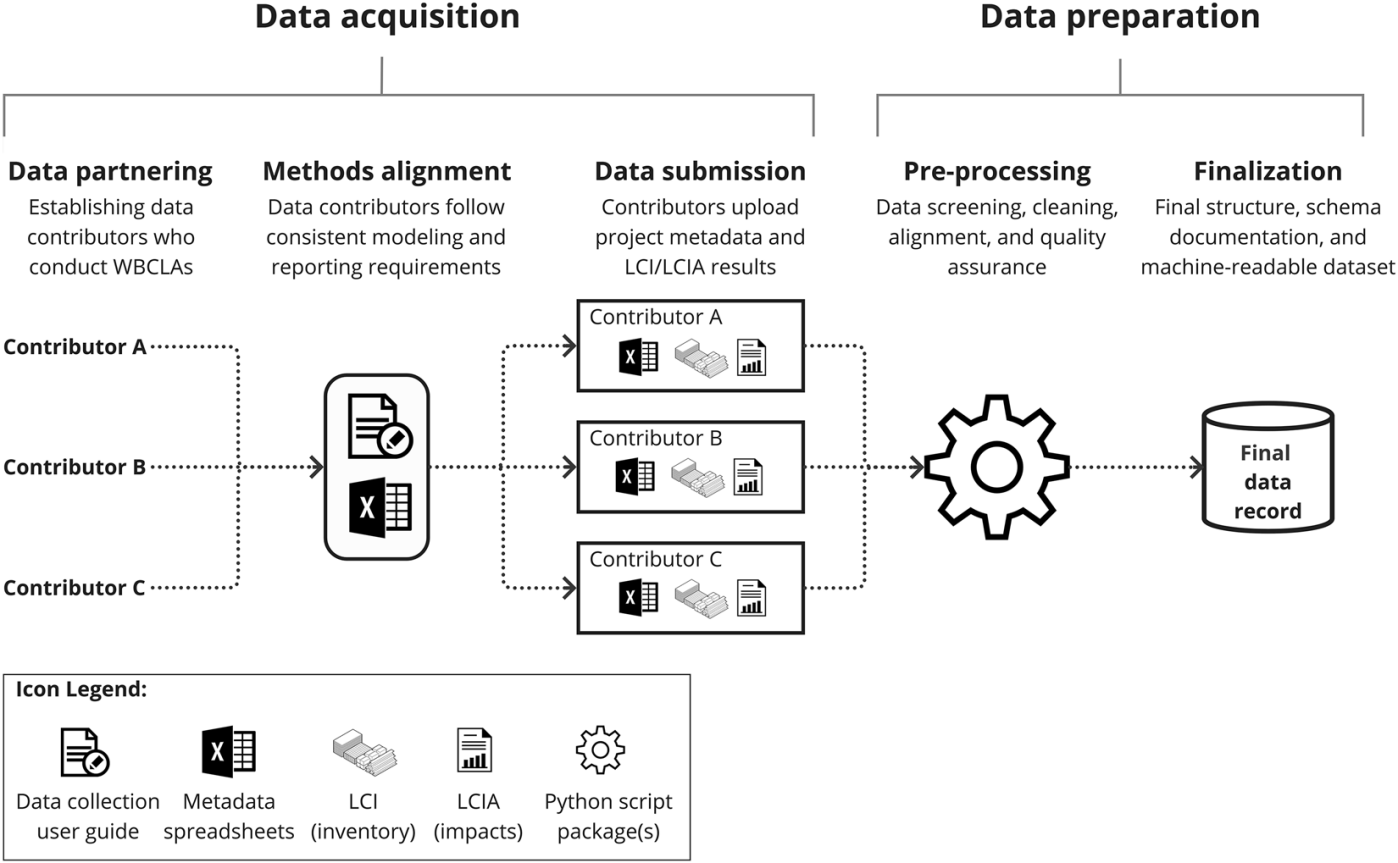
Diagram of the overall data pipeline and methods used to generate the data record.

The data pipeline and methods used to produce the data record are outlined in Fig. 1. This mainly includes two phases: Data Acquisition and Data Preparation. Data Acquisition included steps for data partnering, methodological alignment, and data submission. Data Preparation included steps for data pre-processing and finalization. These phases and steps are described more in the following subsections.

### Data acquisition

As shown in Fig. 1, Data Acquisition began with a data partnering phase to establish a group of data contributors capable of supplying the required data types. This required launching an open call for data and establishing relationships with architecture, engineering, and construction professionals who conduct LCAs of real-world buildings. The open call for data was launched as part of the CLF Benchmark Study v2^52^ and extensive stakeholder engagement with data contributors was conducted to gather feedback on the feasibility of the proposed methods, address data security and privacy concerns, and source all of the project information reflected in the data record. All project data collected was sourced voluntarily from 30 data contributors across North America (primarily architecture and engineering firms). They were required to submit three distinct types of information for each building project they wished to contribute:

- **Project Metadata:**
  - Building Characteristics: General descriptions and physical characteristics of real-world designed or constructed buildings (e.g., project type, project location, construction type, building use, floor area, height, structural system)
  - LCA Modeling Parameters: Documentation of the LCA calculation methods and scenarios used by the modeler for the assessment (e.g., date of analysis, purpose of assessment, physical scope included, reference study period, LCA tool used)
- **Life Cycle Inventory (LCI) Data:** The service life and mass of each individual material used on the building project and included in the LCA, as reported from an LCA tool approved for the study.
- **Life Cycle Impact Assessment (LCIA) Results:** The environmental impacts associated with each material from the LCI, as reported from an LCA tool approved for the study.

A data collection user guide^54^ and a data entry template^55^ were first designed and developed specifically for the data acquisition to ensure consistency and alignment of LCA modeling and reporting methods. The user guide provided guidance and requirements for conducting an LCA with a minimum level of comparability, reporting the required project metadata, and submitting the distinct data types required. The data entry template (Excel spreadsheet) acted as a structured reporting framework for project metadata.

LCI material quantities and LCIA results were required to be reported as exports (Excel files) generated from LCA tools approved for use in the study, namely Tally LCA (version 2018.09.27.01 or later)^56^ or One Click LCA (LEED for US/Canada, TRACI version)^57^. Notably, the user guide and data entry template indicate types of data collected as part of the CLF Benchmark Study V2, which may not be reflected in the data record. Project exclusions, feature exclusions, data flattening (converting multi-layer data into single column/row outputs), and data aggregation (converting wide ranges of numerical or categorical fields into simplified bins) were required to ensure the dataset’s technical validity, preserve project and data contributor anonymity, and protect commercially sensitive information. The key requirements used for project types and LCA modeling of the dataset are summarized in Table 1.

**Table 1:** Data criteria and requirements of the data record.

Category	Requirement Type	Requirement Criteria
Building Projects	Location	North America
	Project types	New construction, renovation, or tenant improvement
	Design phase at time of assessment	Design development (DD) phase or later
	Building use	All types except single-family residential
	Reporting	All project metadata parameters reported in predefined data entry templates
	Other	No limits on the number of projects from each firm; no minimums, maximums, or any requirements for a project’s floor area, height, construction type, structural system, or other design parameters for a project to be included
LCA Models	Reference study period	60 years
	LCA tools allowed	Tally LCA (version 2018.09.27.01 or later) or One Click LCA (LEED for US/Canada, TRACI version)
	Minimum life cycle stages assessed	A1–A3, A4, B4–B5, and C2–C4
	Minimum physical scope included	Structure (including substructure and superstructure) and exterior enclosure for new construction projects. No minimum physical scope for other project types
	Minimum impact categories reported	Global Warming Potential (GWP), Acidification Potential (AP), Eutrophication Potential (EP), Ozone Depletion Potential (ODP), Smog Formation Potential (SFP), and Non-renewable Energy Demand (NRED)
	Reporting	LCA results exported from the allowed LCA tools including full material quantities and impact assessment results
	Other	Additional building elements such as interiors (construction and finishes) were not required, but still submitted for the majority of projects. Building services (e.g., mechanical, electrical, or plumbing systems), sitework (e.g., civil and landscape elements), and equipment and furnishings were not required

The methodological considerations for the criteria in Table 1 were heavily influenced by:

1. the types of data that were available from voluntary data contributors,
2. common building industry LCA modeling practices in North America, and
3. our goal to create a harmonized dataset in alignment with international LCA standards wherever possible, that enables high-resolution comparative analysis across all projects.

While the considerations and the requirements of Table 1 led to certain limitations of the dataset, they were necessary to achieve the quantity and quality of LCA models reflected within it. For example, few data contributors had any single-family residential LCA models and the majority that did modeled them with LCA tools that would have been methodologically inconsistent for comparative analyses. Similarly, the physical scope and life cycle stage requirements reflected what was most commonly and reliably modeled by data contributors at the time of data collection, while also ensuring that direct comparisons of them could be made, given the LCA tools required. While data contributors typically had access to operational energy use data, they rarely performed consistent or comparable assessments of B6 as a life cycle stage. Other criteria were strictly aligned with LCA standards (e.g., reference study period) or created to support comparison making (e.g., design phase).

Finally, data submissions were completed from each data contributor using cloud storage drives. The initial raw data collected included 30 metadata spreadsheets reflecting 325 unique individual projects and 400 LCA model results in total. Although we present the Data Acquisition phase as the first step of our methods, communication with data contributors also extended through the Data Preparation phase. This allowed us to resolve errors or inconsistencies with the data submitted, improve data quality, and ensure the technical validity of the final dataset.

### Data preparation

**Fig. 2:**
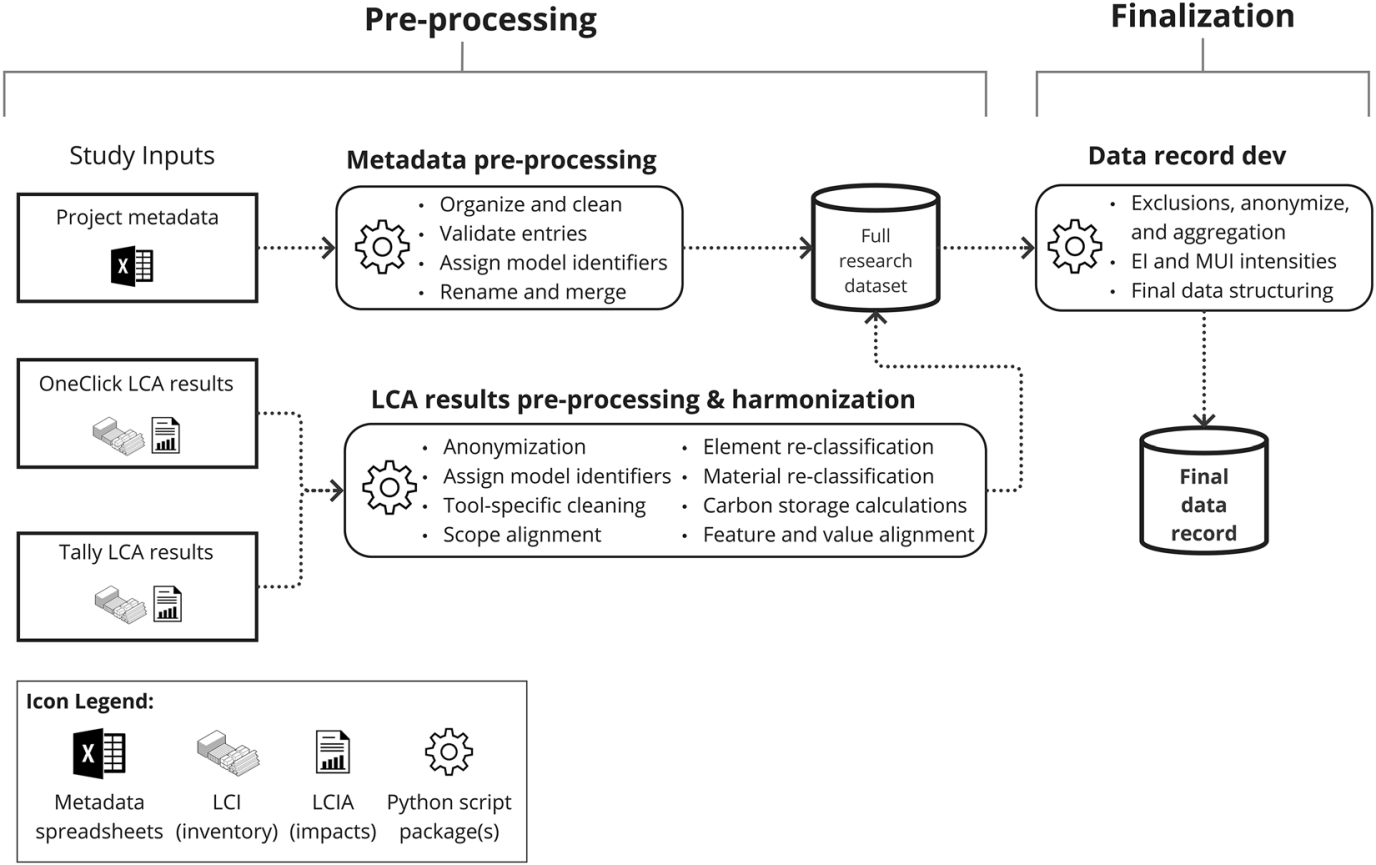
Diagram of the data preparation methods used to produce the data record.

Data pre-processing and finalization, as outlined in Fig. 1, were implemented across all submitted data to address quality issues and increase data harmonization. The detailed process, as outlined in Fig. 2, led to an aligned and machine-readable final data record with the technical validity appropriate for analysis. This work was facilitated using custom code designed and documented for this study with individual workflows for metadata pre-processing, LCA results pre-processing, and data record finalization. Each of these steps is outlined in the following subsections. See the Code Availability section for additional information on the code developed and used.

#### Metadata pre-processing

First, the collected project metadata spreadsheets were manually inspected for completeness, and each project was assigned unique anonymized identifiers prior to entering the data pipeline. Our code then transposed the data and validated consistent column naming, data types, and plausibility of entries in alignment with the data schema. Errors or inconsistencies were manually corrected or automated with defined dictionaries available in the code. In agreement with data contributors, certain indirect project identifiers were binned (bucketed) into intervals based on the distribution of data points and professional judgment to protect project anonymity. Finally, feature names were shortened and renamed in snake case (where multiple words are connected by an underscore). Notably, the feature names used in data preparation and portions of the code reflect the feature names from the raw data collected and not the final data record. Table S1 for matching these differences is available in the Supplementary Information file. This file also contains tables and descriptions for the automated data validation checks performed (Table S2), calculated bins of select features (Table S3), floor area rounding criteria (Table S4), and tables of summary statistic values from figures in the Technical Validation sections of this paper (Tables S5–S14).

#### LCA results pre-processing and harmonization

Tally and One Click LCA are similar tools that can both be used to comply with international building LCA standards such as ISO 21931-1^9^ and EN 15978^58^ but contain significant differences in their background LCI databases, default assumptions for certain life cycle stages and scenarios, and general reporting structures. The LCA results in this study thus required specific pre-processing and harmonization procedures. Data harmonization, particularly concerning building and life cycle assessment data, is ill-defined and often reflects a spectrum rather than a binary. Here we borrow terminology from Cheng *et al*.^59^ to describe what we mean by harmonization. We mainly focused on harmonizing data syntax (i.e., file types), structure (i.e., conceptual schema), and semantics (i.e., the meaning of features and feature groups). While parts of our harmonization were stringent (used identical measures and procedures), the final dataset itself should be considered “flexibly harmonized,” meaning not all data points were created and processed identically, but have been transformed into a common, consistent, and comparable format. As an example, we ensured the harmonization of core LCA modeling criteria (e.g., reference study period, among others) prior to LCA results being submitted to the study but did not restrict LCA models to be generated from a single LCA tool. Other forms of data harmonization are described in the following subsections with efforts made to be clear about their types and extents.

First, LCA results were flattened to single-tab Excel spreadsheets if they weren’t already submitted as such. Next, direct identifiers of the data contributor and their projects were removed and anonymous unique identifiers were assigned (corresponding with identifiers in the project metadata). We then semantically and stringently harmonized feature names and/or their feature groups (the available values within a feature) for TRACI environmental impact categories, life cycle stages, physical scopes, and CSI MasterFormat^60^ divisions. Next, we developed and implemented flexibly harmonized systems for building element classifications, building material classifications, and carbon storage reporting methods. Importantly, we did not attempt to harmonize or validate the background LCI data sources from the LCA tools (e.g., emissions factors of materials). Additionally, we did not attempt to harmonize foreground data or other assumptions used by individual LCA modelers such as the quantities of materials being included, transportation distances and modes selected, replacement rates of materials, selections of certain products, or end-of-life scenarios which all have varying default settings and levels of user control within Tally and One Click LCA. The methods for addressing the building assessment scope and classifications, building material classifications, and biogenic carbon features are introduced below. Note that when we refer to the exact features of the dataset (i.e., columns) in this paper, we use the precise naming of the dataset and list the feature name in italics, often in parentheses after stating its more human-readable name.

### Assessment scope and element classifications

All projects were assessed over life cycle stages A1–A3, A4, B4–B5, and C2–C4. Tally LCA results also included module D, which was maintained in the data record, while One Click LCA results did not. Corresponding inventories and impacts for each life cycle stage were categorized accordingly in the data record (*life_cycle_stage*). Data contributors reported which primary building elements (i.e., the major functional elements that compose a building such as its substructure, superstructure, enclosure, or interiors) were included in their assessments but distinguishing the difference between elements in the LCA results was not always possible. Standards for whole life carbon assessment^61,62^ require the modeling and reporting of LCA impacts by building element categories. While both Tally and One Click LCA implement a tool-specific version of building element mapping, they rely on different schemas (Omniclass Table 21^63^ for One Click LCA and UniFormat II^64^ for Tally LCA), and both required manual assignment or verification by LCA modelers. As shown in our comparison in the Technical Validation section, the accuracy, completeness, and comparability of these default element classification systems were limited and error-prone. Therefore, we reclassified building elements using levels 1–2 of Omniclass Table 21^63^ based on our judgment and other relevant features in the LCA results to create consistency between the tools. For example, if we could identify a building material that was used as a footing or foundation, it was assigned to the building’s substructure. The resulting data record includes assignments for all building elements following Table 2. While this system has limitations, it allows for meaningful analysis and comparisons to be made across tools for the physical scopes included in the assessments. The data record incorporates this system to enable comparison of projects with similar scopes included (*lca_phys_scope*), as well as the LCA results corresponding to those elements (*omniclass_element*).

**Table 2:** Building element classification system reflected in the data record.

Omniclass Number	Omniclass Title (*omniclass_element*)	Abbreviation (*lca_phys_scope*)	Description
21-01 00 00	Substructure	B	Primary at-grade and below-grade construction mainly consisting of slabs-on-grade, foundations, footings, and subgrade enclosure materials.
21-02 10	Shell - Superstructure	S	Primary above-grade structural elements mainly consisting of columns, beams, and framing for floors, roofs, and stairs.
21-02 20	Shell - Enclosure	E	Combination of horizontal and vertical building enclosure elements such as cladding, roofing, doors, windows, insulation, and exterior wall framing.
21-02 30			
21-03 10	Interiors - Construction	C	Non-load-bearing interior construction elements such as interior partitions, ceiling construction, openings, and railings.
21-03 20	Interiors - Finishes	F	Finish elements mainly consisting of wall coverings, acoustic treatments, floor finishes, and ceiling finishes.
NA	Unknown	NA	Any building element or portion thereof that could not be assigned to an above category with reasonable confidence.

### Building material classification

Globally, multiple building material classification standards exist^65,66^. However, across the existing datasets we identified, there is a lack of alignment on the most appropriate system to use when dealing with material quantities or environmental assessment data. In the US and Canadian construction industries, CSI MasterFormat^60^ is a widely used classification system for producing project specifications of building materials. While Tally LCA and One Click LCA both include CSI MasterFormat designations of materials in their output results, they do so only at the top division levels (e.g., concrete, masonry, metals, etc.) while following vastly different naming and classification schemas for individual products and materials. Moreover, no current material classification system in the tools was aligned with the naming conventions commonly used for functionally equivalent products in EPDs. This lack of alignment makes comparing material-level results between tools challenging, and pairing their material quantities to other sources of environmental impact data often impossible.

Therefore, we engineered and implemented a classification system that creates harmonization between the LCA tools and mirrors the naming conventions commonly used for functionally equivalent products in current EPDs and PCRs, where applicable. The resulting system includes two new material classification levels: *mat_group* which classifies similar materials by 22 general groups (e.g., “Concrete”), and *mat_type* which classifies over 122 functionally equivalent—or functionally similar—types of products within a group (e.g., “Ready mix concrete LW 3000 psi”). Notably, the *mat_type* classifications may include multiple materials that are not fully equivalent and descriptions of the products included under each type are listed in the material_definitions.xlsx file of the data record. Associated (typically approximate) CSI divisions and product category rules are also listed per *mat_type* in the file for reference. Other classifications native to each LCA tool were also maintained in the data record including top divisions of CSI MasterFormat (*mat_csi_division*), two classifications unique to Tally LCA results (*tally_revit_building_element* and *tally_material_group*), and two classifications unique to One Click LCA results (*oneclick_omniclass* and *oneclick_resources_type*). Select material mappings were then used to further refine and enhance the accuracy of our prior building element classifications.

### Biogenic carbon

Biogenic carbon refers to carbon sequestered from the atmosphere during biomass growth, stored in bio-based materials during their use phases, and released back into the atmosphere due to decomposition or combustion^67–69^. Biogenic carbon can be reported in a building LCA using one of two fundamentally different approaches: as an emission (i.e., GWP-bio) or an inventory metric (i.e., stored carbon). Currently, the North American versions of Tally and One Click LCA represented in this dataset use different methodologies for quantifying and reporting biogenic carbon. Neither of the versions of the tools used in this dataset report biogenic carbon emissions (i.e., GWP-bio) separately from fossil emissions (i.e., GWP-fossil) in a consistent manner, making comparison across models impossible. For example, the same building modeled in both tools may potentially report net negative emissions in one and positive emissions in the other^69^.

To increase comparability across building models, we excluded the uptake and emissions of biogenic carbon when reporting GWP totals and intensities across all models. GWP totals and intensities reported in the data record reflect only GWP-fossil per EN 15804 + A2^70^, with “negative emissions” or carbon sequestered during plant growth excluded from A1-A3 impacts. The amount of biogenic carbon that enters the system boundary through the use of a bio-based material is reported separately for each project and material as an inventory metric (*inv_stored_carbon*) in kgCO_2_e. This is the default methodology for One Click LCA for LEED (TRACI) models^71^, but it required custom calculations to enable a comparable output for Tally LCA results. For all Tally models, this was achieved by estimating the carbon content per kilogram of material as specified by EN 16449^72^ and referenced by ISO 21930^73^ and EN 15804 + A2^70^. The core calculation used is as follows:

$${P}_{{CO}2}=\frac{44}{12}\ast {cf}\ast \frac{{\rho }_{\omega }\ast {V}_{\omega }}{1+\frac{\omega }{100}}$$

Where

*P*_*CO2*_ *is the mass of biogenic carbon oxidized as carbon dioxide when emitted from the product system into the atmosphere*

*44/12 is the ratio between the molecular mass of CO*_*2*_ *and C molecules*

*cf is the carbon fraction of woody biomass (oven dry mass), may use 0.5 as a default value*

*w is the moisture content of the product (e.g. 12%, 6%)*

*ρ*_*w*_ *is the density of woody biomass at that moisture content (kg/m3)*

*V*_*w*_ *is the volume of solid wood product at that moisture content (m3)*

For engineered wood products, wood volume content *V*_*w*_ = *VP* * % wood or bio content in the full product, where *VP* is the gross volume of the wood-based product

Since Tally’s background data is reported in kilograms, not volume, the carbon quantity will be identical across wood species on a mass basis. Therefore, the following equation was used to convert the mass of the delivered wood product into a dry weight based on a specific moisture content:

$${P}_{{CO}2}=\frac{44}{12}\ast {cf}\ast m\ast \frac{1}{1+\frac{{MC}}{100}}\ast {bc}$$

Where

*P*_*CO2*_ *is the mass of biogenic carbon stored in the product or material during its useable life*

*44/12 is the ratio between the molecular mass of CO*_*2*_ *and C molecules*

*cf is the carbon fraction of woody biomass (oven dry mass), 0.5 used as a default value*

*m is the mass of the product in kg*

*MC is the moisture content of the product (e.g. 12%, 6%)*

*bc is the biogenic content of the product measured in % wood or fiber based on mass if the product is a composite material*

The carbon fraction of woody biomass used a consistent default value (0.5), and the moisture content of the wood product was based on specific product information such as an EPD or relevant product literature representing typical production. This equation may be used for solid wood, such as dimensional lumber, or composite materials of wood or fiber if the percent bio-based content per functional unit is known. For all composite or engineered wood products, the percent bio-based content was derived from a relevant EPD based on mass. To calculate the moisture content of an assembly with two or more materials, a weighted average was used based on mass. Within the code repository (see Code Availability section), a table is provided (“stored_carbon_database.xlsx” within the references folder) that contains the product-specific moisture contents, percent wood by mass, and data sources that we used per material for the quantification of carbon storage. The resulting carbon storage (*inv_stored_carbon*) of each bio-based material for life cycle stages A1-A3 is reported in kgCO_2_e^74^.

### Finalization

After pre-processing and harmonization, the data were merged into a full research dataset as shown in Fig. 2. The full research dataset contained sensitive project information that could not be distributed per agreements with data contributors and project types that did not meet the data collection requirements. Producing the final dataset thus required additional project screening, quality assurance, anomaly assessments, anonymization, and final data cleaning which was handled primarily through code. Any projects or project data that did not meet the study requirements (see Table 1) such as baseline design models, multiple LCA iterations of the same projects, and/or identifiably erroneous models were excluded. This resulted in a total of 292 LCA models retained in the dataset from the original 400 collected. Portions of LCA impacts that were not modeled with consistency or sufficient resolution (services, sitework, and equipment and furnishings) were also excluded. All other projects, including identified extreme data points for total MUI and ECI, were retained in the dataset if they could not be proven erroneous in terms of their data structure and/or evident LCA modeling methods applied. Additional context on outliers is presented in the Usage Notes section. Per requests from data contributors, all floor areas were rounded according to the criteria in Table S4 (see Supplementary Information document).

Material mass, environmental impacts (GWP, EP, AP, SFP, ODP, and NRED), and their respective intensities and totals were then calculated for each project and material. When MUI and EI intensities were calculated per unit floor area of new construction projects, we provided two separate normalizations: one using total constructed floor area (CFA) and another for total gross floor area (GFA). For this dataset, CFA includes the floor area of any attached or integrated parking components whereas GFA (as defined by IPMS 2^75^) is effectively the difference between the building’s constructed floor area and its parking area. These two methods can lead to large differences in intensity results based on the size of parking components included in projects. Furthermore, there is a lack of agreement in the design industry and among existing studies on the most appropriate method to use. For renovation projects (major, minor, and tenant improvements), impact intensities based on floor area were normalized by the combination of renovated floor area and added floor area (i.e., *bldg_added_GFA* + *bldg_renovated_GFA*). Impact intensities were also calculated based on the number of building occupants and residential units for projects where applicable.

The final data structure and feature-naming convention of the data record was informed by the Embodied Carbon Harmonization and Optimization (ECHO) Schema V1.0^76,77^ which is a North American effort to create alignment across building LCA reporting efforts and databases. Direct feature mappings to this system are provided in the data_glossary.xlsx file of the data record, where applicable. Finally, data contributors were consulted on the structure, content, accuracy, and level of data anonymization in the dataset and informed consent was obtained from all contributors before publication.

## Data Records

The full dataset is available on Figshare^78^ and this data descriptor corresponds to version 2 of the Figshare repository. The dataset contains four files:

- **buildings_metadata.xlsx** includes all project metadata and LCA parameters for every project associated with a unique index number to cross-reference across other files. This also includes various calculated summaries of LCI and LCIA totals and intensities per project.
- **full_lca_results.xlsx** includes LCI and LCIA results per material and life cycle stage of each building project.
- **data_glossary.xlsx** identifies and defines each feature of the dataset including its name, data structure, syntax, units, descriptions, and more.
- **material_definitions.xlsx** a full list of material groups, types, and descriptions of what they include.

### Data structure and contents

The dataset is primarily composed of two separate files that can be merged or joined using common keys (*project_index*), which are assigned to each project to facilitate a wide range of uses and types of analysis. The first is the buildings_metadata.xlsx file, which is structured so that each row of data reflects a single project. It contains 72 features organized by feature types including site context, building design, structural design, LCA methods, and calculated summaries. The second is the full_lca_results.xlsx file, which is structured in a way that mimics the non-aggregated model output results of Tally LCA and One Click LCA. It contains 21 features organized by feature types for LCA classifications, LCI results, LCIA results, and calculated summaries. In this format, each row of data reflects a single material and life cycle stage from an individual project. This allows for high-resolution and flexible analysis but may include seemingly duplicate entries when the same material or element was modeled in multiple different instances across a single project (e.g., a project with three different walls which were all composed of 4000 psi concrete will display three separate times in each life cycle stage for that project). Similarly, each material is reported per life cycle stage regardless of whether it has environmental impacts or mass associated with it. The mass of materials is only reported under life cycle stages A1–A3 for new materials, and B4 for replaced materials. When rows contain “0” values for mass or impacts across different life cycle stages, it can be due to the materials being existing or salvaged, having no actual impacts, or due to inconsistencies or errors in the LCI background data within LCA tools which we did not attempt to address. For further information on this format, see Usage Notes.

Two additional files are provided to assist users in understanding the dataset. A full data glossary (data_glossary.xlsx) defines and describes each feature of the dataset including the files they are used in and their feature types, names, descriptions, data types, units, references, measurement types, usage notes, and equivalent ECHO V1.0 mappings where applicable. Custom feature groups—those that follow uncommon or non-standardized conventions—are further detailed on the 2nd tab of the data_glossary.xlsx file. The last file (material_definitions.xlsx) contains names and descriptions of all material groups and types that the dataset includes. Associated CSI divisions and product category rules for each material type are noted within it for reference, where applicable.

Following Röck *et al*.^43^, features are distinguished by measurement type within the data_glossary.xlsx file based on their source:

- **Primary** features and their values were directly reported by data contributors.
- **Secondary** features and their values were computed, inferred, or engineered by the authors based on data provided by contributors or best judgment.

All data types are either string (predefined, open, or binned) or float (whole or decimal numbers) and each contains units where applicable.

## Technical Validation

The validity of the dataset is described here first in terms of the methods we used to collect, pre-process, harmonize, and otherwise manipulate the dataset followed by tests for data applications and consistency.

### Methodological Validity

The resulting dataset contains only 7% and less than 1% total missing values (NULLs) for buildings_metadata.xslx and full_lca_results.xlsx files, respectively. The majority of missing data points for buildings_metadata.xlsx relate to features that were less available and less prioritized during the data collection process, such as the average R-values of walls and roofs, thermal envelope areas, and window-to-wall ratios. With the exception of impact intensities per occupant and residential units, all calculated summaries are 100% complete.

Before building element reclassification, the raw LCA results data contained many “undefined” building elements. These represented an average of 23% of each project’s total GWP from life cycle stages A–C not being assigned to a building element. After completing our building element reclassification, this average was reduced to less than 1% of each project’s total GWP remaining undefined. Accordingly, the average A–C GWP impacts of building elements tended to increase in our dataset after the reclassification, with Interiors (combination of construction and finishes) seeing the largest increase (+23%), followed by Shell-Enclosure (+19%), and Shell-Superstructure (+6%). Average Substructure impacts were the only ones to decrease (−5%). These values were found to be consistent with other studies in the Environmental Impacts section below. These tests can be performed or further explored using *oneclick_omniclass* and *tally_revit_building_element* features which display the original building element classifications native to the raw LCA results collected.

Building material classification resulted in a condensation and simplification of the dataset. The raw LCA results collected contained over 1,500 material names. Many of these names were duplicates, near-duplicates, or otherwise incomparable to each other due to semantic classification differences across LCA tools and versions. The resulting data record after material mapping includes materials corresponding to 117 unique material types (*mat_type)* of the 122 types originally developed in the code. These material types are categorized under 22 material groups (*mat_group*). The material group “Other” reflects materials that are effectively unclassified. These materials account for 0–3.8% of each project’s GWP (average value = 0.23%), and between 0–7% of each project’s mass (average value < 1%).

Floor areas were rounded per agreement with data contributors as described in the Methods section. Since the environmental impact and material use intensities provided are predominantly based on floor areas, this rounding affected the accuracy and specificity of these values. These impact intensity differences due to rounding were minor and ranged from approximately −3 to +1% with median and mean differences of less than 0.02% in either direction.

All 30 data contributor companies reviewed the accuracy and completeness of their projects throughout the Data Acquisition process and again for the final dataset. Ultimately, each contributor provided a final review and approval of their project data. Known or discoverable errors were resolved accordingly through metadata re-entries or LCA results resubmissions. These reviews applied to both the raw data submitted by contributors and the results of data manipulations from our methodology. Compared to the existing datasets we examined, this form of technical validation was unique to our study and allowed for feedback, iteration, and refinement of all data collected throughout the project.

Throughout our methodology, we performed analyses and explorations to detect errors and outliers, test the data structure, identify correlations, explore GWP factors of the LCA tools, and assess the MUI, LCI, and LCIA results of the building projects against their metadata. In the following section, technical validation is presented with a specific focus on data applications and data consistency.

### Data applications and consistency

Intended and assumed applications of the dataset include, but are not limited to, analyzing the EIs and MUIs of building projects with respect to different project features. To broadly validate the dataset and highlight its applications, several expansion studies have been conducted that we encourage readers to explore:

- Shen *et al*.^79^ performed exploratory data analysis that validated the datasets applicability for sophisticated statistical use cases. This included advanced methods such as bivariate analysis which explored correlations and differences between attribute pairs or groups using statistical tests such as correlation analysis, ANOVA, and post-hoc analyses. Feature engineering techniques extended the research to multivariate dimensions, identifying attribute impacts and global correlations.
- Ashtiani *et al*.^80^ examined the material use and embodied carbon intensities of buildings within the dataset with special focus on the influence of the use of material groups and types across various building typologies and project features. This included average contribution assessments across buildings and a visualization tool (https://wblca-benchmark-v2-mui-eci.lifecyclelab.org/) to make highly specific comparisons at the building and material levels.
- Chafart *et al*. produced an additional visualization dashboard (https://wblca-benchmark-v2.lifecyclelab.org/) enabling broad comparisons of EIs across projects.
- Benke *et al*.^81^ used and enriched a subset of projects in the dataset to establish recommended EC limit values to be used in building-scale LCA policies and programs.

Across each of these efforts, the validity and applicability of the dataset were shown to offer strong capabilities for researchers, designers, policymakers, and others in the building industry to assess building impact trends, set limits or targets, and identify aspects of high and low impact projects using the dataset.

Furthermore, the validity, variation, and consistency of the dataset are tested in the following subsections and, where possible, comparisons are made to similarly scoped datasets and studies. Tables of specific summary statistic values from each of the following figures are available in the Supplementary Information file (Tables S5–S14), which were generated from the Tableau Desktop software. For box and whisker plots, Tableau Desktop utilizes the Tukey method^82^ of quantification. This results in upper and lower “hinges” (effectively medians of the upper and lower 50% of data points) in place of pure quartiles. For large datasets like the one presented in this study, these differences are negligible and for simplicity, we still refer to the range between the lower and upper hinges as the “interquartile range”. For each figure that follows, material use and impact intensities were normalized by CFA.

#### Data completeness and coverage

The general coverage, completeness, and distribution of projects and LCA models can be assessed using various dataset features. Counts of project types (*bldg_proj_type*) and physical scopes included per project (*lca_phys_scope*) are shown in Table 3. The majority of buildings are new construction projects (n = 243, 88%) and they include a minimum of Substructure, Shell-Superstructure, and Shell-Enclosure building elements (BSE). Of these projects, 80 (33%) include no interiors (BSE), nine (4%) include partial interiors (BSEC or BSEF), and 154 (63%) include full interior elements (BSECF). Major Renovation (n = 25), Minor Renovation (n = 17), and Tenant Improvement projects (n = 7) contain various physical scopes based on the type and extent of the construction work involved.

**Table 3:** Count of projects by their respective project types and physical scopes included in the assessments.

Project Type (*bldg_proj_type)*	Physical Scope Included (*lca_phys_scope)*									
	BSE	BSEC	BSEF	BSECF	CF	ECF	SCF	SEC	SECF	Totals
New Construction	80	6	3	154						243
Major Renovation	6			15	1		1		2	25
Minor Renovation				7	3	1	2	2	2	17
Tenant Improvement					6	1				7
Totals	86	6	3	176	10	2	3	2	4	292

Abbreviations include B = Substructure, S = Shell - Superstructure, E = Shell - Enclosure, C = Interiors - Construction, and F = Interiors - Finishes.

**Fig. 3:**
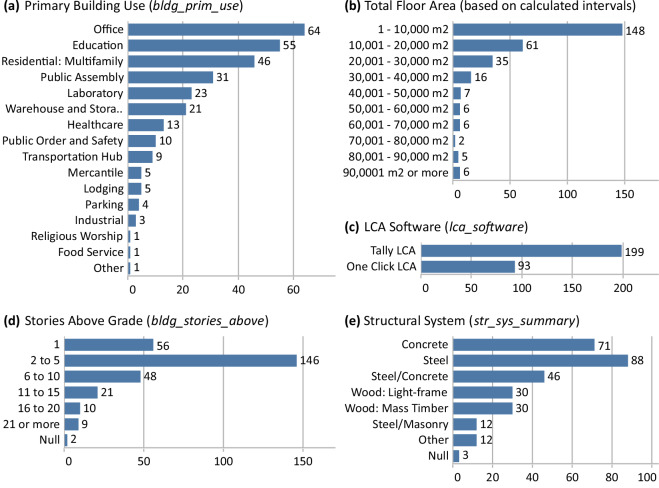
Count of projects based on primary building use type (**a**), total floor area by floor area range (**b**), LCA software used (**c**), stories above grade (**d**), and structural system summary (**e**). See Tables S5–S9 of the Supplementary Information file for specific values from this figure.

The distribution of other metadata features is shown in Fig. 3. The buildings are predominantly non-residential (84%). Projects range in geometry but are mostly modestly sized with 51% having floor areas of 10,000 m2 or less and 69% being less than 5 stories above grade. Steel, concrete, and steel/concrete hybrid structural systems make up over 70% of the projects represented. Just over 2/3 of the LCA models were conducted using Tally LCA.

#### Environmental impacts

**Fig. 4:**
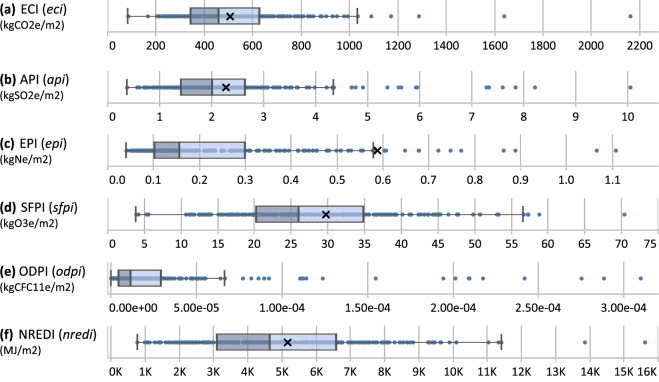
Boxplot distributions of impact intensities for ECI (**a**), API (**b**), EPI (**c**), SFPI (**d**), ODPI (**e**), and NREDI (**f**) for new construction projects (n = 242) of life cycle stages A–C. Note multiple X-axes and units are displayed. For visibility, extremely high data points are hidden from the chart for EPI (n = 6), SFPI (n = 2), ODPI (n = 7), and NREDI (n = 2). See Table S10 of the Supplementary Information file for summary statistic values from this figure. Intensities are based on CFA normalization.

**Fig. 5:**
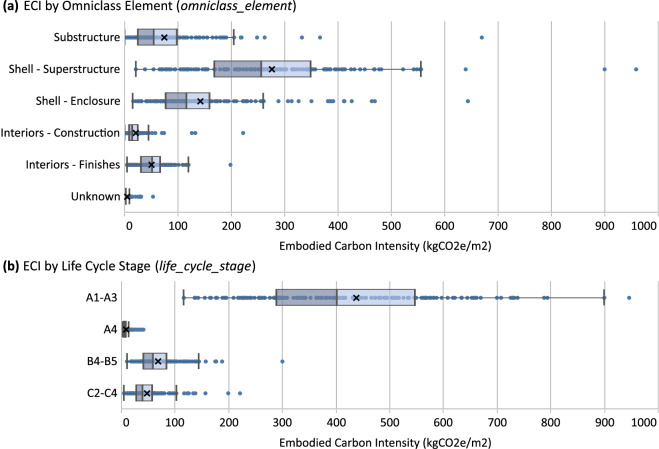
Boxplot distributions of ECI for Omniclass elements (**a**) and life cycle stages (**b**) for new construction projects including BSECF scope (n = 154) and life cycle stages A–C. For visibility, extreme data points over 1000 are hidden from the chart (n = 1 in Shell - Superstructure, n = 3 in A1-A3). See Tables S11, S12 of the Supplementary Information file for summary statistic values from this figure. Intensities are based on CFA normalization.

Projects can be assessed using various environmental impact categories and calculated summaries. Here, we present the distribution of EI intensities of the dataset. This can be performed for all projects and impacts as shown in Fig. 4, or for specific EI intensities and dataset features, such as ECI results by Omniclass building element and life cycle stage as shown in Fig. 5. For the box and whisker plots shown, the dividing box line indicates the median, the “x” indicates the mean.

In Fig. 4, our dataset shows ECIs of new construction projects for life cycle stages A–C ranging from 84–2160 kgCO_2_e/m^2^, an interquartile range of 343–628 kgCO_2_e/m^2^, and mean and median values of 505 and 461 kgCO_2_e/m^2^, respectively. The mean values of similarly scoped studies and datasets (i.e., when they are limited to structure, enclosure, and varying degrees of interiors, life cycle stages A–C, and exclude single-family residential) all fall within our dataset’s interquartile range. These included mean kgCO_2_e/m^2^ values from Röck *et al*.^20^ as quantified by the authors from available data (429 kgCO_2_e/m^2^), TAF *et al*.^83^ when averaged across similar use types (415 kgCO_2_e/m^2^), and OneClick *et al*.^84^ (468 kgCO_2_e/m^2^) which also required averaging across use types.

Limited studies exist to make equivalent comparisons of other impact intensities to our dataset. Bowick *et al*.^85^ investigated ten multifamily residential projects in British Columbia of similar physical scopes and life cycle stages. When looking only at multifamily residential buildings, our dataset’s median values for ECI (373 kgCO_2_e/m^2^), API (1.71 kgSO_2_e/m^2^), EPI (0.11 kgNe/m^2^), and NREDI (3641 MJ/m^2^) all fall within the upper and lower ranges of their study. Our median SFPI (21.4 kgO3e/m^2^) fell just below their range, representing a 45% decrease compared to their median (33.9 kgO_3_e/m^2^). The most notable difference was the ODPI values. Our median ODPI for multifamily residential projects (9.12e-06 kgCFC_11_e/m^2^) was significantly larger than theirs (2.74e-06 kgCFC_11_e/m^2^) representing an increase of 108%. We examined the influence of the LCA modeling tool and found that our median ODPI for Tally LCA models (5.38e-06 kgCFC_11_e/m^2^) was closer to the range of Bowick *et al*. and extreme outliers for ODPI in our dataset were generated from One Click LCA models. Similar differences were observed for EPI and NREDI values between Tally LCA and One Click LCA models in our dataset. As the Athena Impact Estimator LCA tool was used in Bowick *et al*., these variations appear more likely related to background datasets of different LCA tools being used rather than differences in actual project emissions.

As shown in Fig. 5, Shell-Superstructure represents the largest range of building element ECIs (20–1615 kgCO_2_e/m^2^) in our dataset and the greatest share of total ECI on average (275 kgCO_2_e/m^2^). It’s followed by average ECIs of Shell - Enclosure (141 kgCO_2_e/m^2^), Substructure (75 kgCO_2_e/m^2^), Interiors - Finishes (51 kgCO_2_e/m^2^), and Interiors - Construction (21 kgCO2e/m^2^). ECI impacts from life cycle stages A1–A3 far outweighed those of other stages, ranging from 115–1929 kgCO_2_e/m^2^ with a mean of 437 kgCO_2_e/m^2^ compared to the means from stages A4 (8 kgCO_2_e/m^2^), B4–B5 (69 kgCO_2_e/m^2^), and C2–C4 (48 kgCO_2_e/m^2^).

While ECI is more widely studied, making direct comparisons of building elements is still challenging due to differences in LCA modeling and reporting methods. Here, Röck *et al*.^20^ provide data that enables a reasonable, albeit limited, comparison. Notably, their dataset includes only European building projects, uses a classification system based on BB-CI/SfB^86^ which is not directly comparable to Omniclass, and relies on data generated from different LCA modeling tools and methods. Still, it is one of the only datasets with LCA results presented by building elements. We first multiplied their provided annualized ECI values by a 60-year reference study period to harmonize with ours, isolated use types to non-residential, and combined our Interiors - Finishes and Interior - Construction into a single element group representing all interiors similar to their “Internal” classification. We isolated an equivalent sample of buildings from our dataset and found our substructure, superstructure, and enclosure median values fell within the interquartile range of their dataset and vice versa. This comparison alone shows reasonable consistency but there were significant percent differences between median values, particularly for interiors. The differences of our median values compared to theirs included Substructure (+30%) Shell - Superstructure (+18%), Shell - Enclosure (+19%), and Interiors (−57%). We repeated this comparison using mean kgCO_2_e/m^2^ values found differences including Substructure (−24%), Shell - Superstructure (+10%), Shell - Enclosure (−6%), and Interiors (−62%). Overall, we found reasonable consistency between Substructure and Shell-Enclosure elements while our dataset shows consistently higher ECIs for Shell-Superstructure, and lower ECIs for interiors. As our study did not require interiors to be included in assessments, we received varying degrees of completeness for interior elements which likely explains the difference for interiors. Differences in Shell - Superstructure may be attributable to actual variations in building design and construction practices, the data samples collected, or other methodological differences discussed above.

We limited comparisons to other studies for life cycle stages to A1–A3 as these impacts are less dependent on differences in LCA modeling methods for later stages such as transportation distances and modes (A4), replacement rates of materials (B4), and end-of-life scenarios (C3–C4). Accordingly, we found that the mean A1–A3 ECI value of our dataset (437 kgCO_2_e/m^2^) fell within the interquartile ranges of Röck *et al*.^20^ using the same criteria above (325–480 kgCO_2_e/m^2^) and Simonen *et al*.^18,19^ when limited to equivalent physical scopes (274–534 kgCO_2_e/m^2^).

#### Material use

**Fig. 6:**
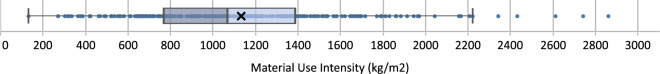
Boxplot distribution of MUI of new construction projects. For visibility, extremely high data points are cropped from view (n = 1). See Table S13 of the Supplementary Information file for summary statistic values from this figure. Intensities are based on CFA normalization.

**Fig. 7:**
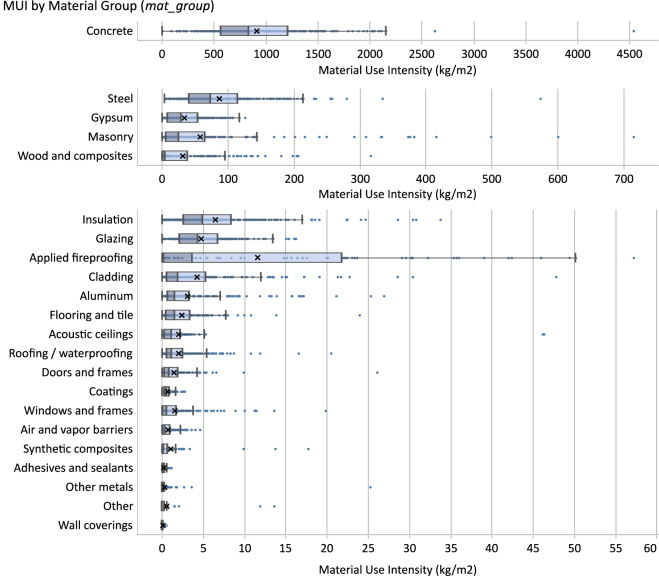
Boxplot distributions of MUI of material groups for life cycle stage A1-A3 of new construction projects. Results are ordered by descending medians. Note that three X-axis scales are used for readability. See Table S14 of the Supplementary Information file for summary statistic values from this figure. Intensities are based on CFA normalization.

The MUIs of new construction projects are displayed for entire buildings in Fig. 6 and per material groups (*mat_group*) in Fig. 7. Here, both figures limit the calculation of MUI to the mass associated with the initial installation of materials in A1–A3.

The MUIs of new construction projects in Fig. 6 range from 130–4907 kg/m^2^ in extreme cases with an interquartile range of 769–1388 kg/m^2^, a median of 1071 kg/m^2^, and a mean of 1135 kg/m^2^. Our total MUIs were highly consistent with similar datasets. We compared our data to Guven *et al*.^26^ by filtering their dataset to exclude all single-family residential, which resulted in an interquartile range of 743–1246 kg/m^2^ and a median of 1022 kg/m^2^ (5% difference in the median values). Similarly, we compared our data using the available MUIs from the deQo dashboard (https://www.carbondeqo.com/database/graph) by filtering to USA projects which returned an interquartile range of 724–1334 kg/m^2^ and a median value of 942 kg/m^2^ (13% difference in the median value).

As shown in Fig. 7, the top 5 largest MUIs by median values in our dataset were Concrete, Steel, Gypsum, Masonry, and Wood and Composites. Notably, the MUIs in our dataset, particularly for structural materials, were heavily influenced by the structural systems used on the projects (*str_sys_summary*). While Fishman *et al*. provided a dataset^28^ of comparable MUI ranges for multiple similar material groups, there are limited comparisons that can be made using equivalent structural system types. We first excluded single-family residential buildings and used their dataset to compare only the core material of the structural system itself (e.g., the concrete MUI of a concrete structural system). This was only possible for structural systems corresponding to their groupings of “reinforced concrete”, “stee”l, and “timber” which corresponded to our groups for Concrete, Steel, and the combination of Wood: Mass Timber and Wood: Light-Frame. The results showed strong consistency between the datasets with the percent differences of our median values compared to theirs including concrete (+13%), steel (−4.3%), and wood (+30%). The larger difference for wood may likely be attributable to different methodologies for dealing with biogenic carbon.

### Limitations

The foundational components of our dataset were LCA models of building projects and their corresponding results. These results can vary in accuracy depending on the goal, scope, purpose of the assessment, methods, modeling assumptions, and skill of the LCA modeler. Additionally, different modeling standards, guidelines, LCA tools, and datasets used in assessments can cause significant differences in results. Reported EIs can also differ between the design and as-built stages owing to changes that occur during the construction process. To our knowledge, none of the submitted models represented measured material quantities from a job site, even when the model endeavored to represent as-built conditions. While efforts were made to conduct quality assurance and harmonize all data produced and collected for this dataset, it is inherently difficult to verify the accuracy of LCA models that were externally developed. While the data record indicates precise environmental impacts, they should be viewed only as estimations of the real-world emissions of constructed buildings.

Due to the challenges of data collection and the variability in LCA modeling, the completeness and accuracy of all LCA models used for this dataset cannot be verified. All models in the dataset were design models, produced by project architects, engineers, and consultants using their professional judgment to assess the design intent. The scope of the data collected was also limited and focused only on the embodied impacts from life cycle stages A1–A3, A4, B4–B5, and C2–C4 for temporal boundaries, and excluded sitework, services (mechanical, electrical, and plumbing), and equipment and furnishings in their physical boundaries. Additionally, all project metadata reported as part of the data collection process relied on manual inputs by data contributors.

Several efforts were made to validate the collected metadata for the dataset. We asked clarifying questions to data contributors, cross-checked the data against other information provided for the project, compared them to other projects in the dataset, and/or used our professional judgment to help ensure that each value provided was plausible for the given building project, if not confirmed. Still, the final project metadata in the data record cannot be fully verified in terms of real-world accuracy or specificity and should be treated as such. Similar processes were carried out to spot-check outliers and potential omissions in building element scope and material assignments.

Data collection was predominantly tailored towards new construction projects, but multiple renovation projects were submitted by data contributors and included in the dataset. Consistent LCA modeling methods and metadata reporting criteria are less established and agreed-upon for these project types within current LCA standards and the design industry. Accordingly, the data collection process contained ambiguities regarding how these project types should be reported (e.g., whether the structural design criteria should refer to the existing building or the new renovation work). Quality assurance could not be performed for renovation projects (major, minor, and tenant improvements) to the same extent as was done for new construction projects in the dataset. They should be considered limited in their application and use. Lastly, we did not evaluate how representative the dataset is in terms of historical, current, or future North American construction as a whole.

## Usage Notes

Each dataset feature includes individual usage notes in the data_glossary.xlsx file, where applicable. These usage notes include objective notes and subjective recommendations for the features based on our insights from data collection, preparation, and assumed common use cases. Additional usage notes for specific topics are included in the following subsections.

### GitHub repository

The dataset is mirrored on a public Github repository (https://github.com/Life-Cycle-Lab/wblca-benchmark-v2-data). The Github provides an additional repository for the dataset and may be extended or modified in the future to include more building projects, additional project metadata, or increased resolutions of LCI or LCIA data.

### Data structure for full_lca_results.xlsx

As described in the Data Records section, users may be unfamiliar with the structure of data in the full_lca_results.xlsx file, which replicates the way Tally LCA and One Click LCA generate model output results. It is, however, one of the most valuable aspects of the dataset and allows for high-resolution analysis and comparison making. We recommend users familiarize themselves with its data structure prior to attempting analysis. For seemingly duplicate entries–when all feature values of a row are identical–users may prefer to aggregate those rows of data per project. When rows of data have exclusively “0” values, users may prefer to delete or ignore them. While it’s fully possible to open, process, and analyze this data natively in Excel, we recommend the use of coding to leverage its full potential. In Ashtiani *et al*.^80^, open-source code for the analysis of ECIs and MUIs was provided. Though this code was developed for specific research questions and purposes, it may serve as a helpful example for users working with the full_lca_results.xslx file through code for the first time.

### Project type and scope

Users should pay particular attention to project types, physical scopes, and life cycle stages (among others) as not all projects in the dataset are reasonably comparable or functionally equivalent.

Project types (*bldg_project_type*) and their respective groups (New construction, Major renovation, Minor renovation, and Tenant improvement) are only reasonably comparable across identical types as the amount and extent of actual construction work can vary widely across types. Additionally, project types other than New Construction were not the focus of the data collection and data preparation processes and should be treated with caution. See the Limitations section for additional context.

It is important to distinguish between the building elements that were reported by contributors as included in the assessment using *lca_phys_scope* and the actual inventory or impacts of those building elements in the full_lca_results.xlsx file which can be selected and filtered using *omniclass_element*. For example, projects that reported including ‘BSE’ (Substructure, Shell-Superstructure, and Shell-Enclosure) in the assessment often contain small amounts of materials and impacts from other elements such as the Interiors-Construction, Interiors-Finishes, and Unknown element categories. We recommend prioritizing the use of *lca_phys_scope* when comparing data at the project scale. In contrast, *omniclass_element* is useful for isolating and comparing the impacts of individual elements but may omit impacts at the project level. Furthermore, our building element reclassification method had limitations. It was particularly challenging to distinguish and uniquely classify between two types of structural elements (Substructure and Shell-Superstructure) and two types of interior elements (Interiors-Construction and Interiors-Finishes). Users may find it more meaningful to combine the two into simplified and respective bins for “Structure” and “Interiors”.

Lastly, inventories and impacts can be filtered and compared by their respective life cycle stages. Importantly, all results from Tally LCA include module D, whereas those from One Click LCA do not. This discrepancy can be addressed by comparing only results by certain tools (*lca_software*), isolating specific life cycle stages (*life_cycle_stage*), or both.

### Normalization metrics

Select summaries of material use and impact intensities are included in the buildings_metadata.xlsx file. These intensities are largely based on floor area normalizations which are common within the building industry. For new construction projects, EI and MUI intensities based on floor area were calculated for life cycle stages A–C and normalized using both constructed floor area (*bldg_cfa*) and gross floor area (*bldg_gfa*). Notably, all floor areas were rounded as described in the Methods section. Users can also quantify different intensities entirely using any continuous data feature.

For major renovations, minor renovations, and tenant improvement projects, impact intensities based on floor area were calculated for life cycle stages A–C and normalized by the combination of renovated floor area and added floor area (i.e., *bldg_added_GFA* + *bldg_renovated_GFA*). These calculated summaries will remain unchanged based on the metric selected. There is less industry-wide and academic research agreement on the type of normalization to use for these project types. Users should be aware they are quantified differently than new construction projects.

Impact intensities per occupant and residential units were also included in the buildings_metadata.xlsx file where applicable. Notably, the type of occupancy provided by data contributors is based on their applicable building and fire codes. Thus, occupancies reported are effectively the maximum allowable occupants of the buildings for fire safety, and not the average building occupants or full-time equivalent occupants of the buildings.

### Missing information

To avoid ambiguity in the data record, missing (empty) values were addressed using the following notations for both categorical and continuous variables.

- **“0” = Zero**. A “0” value was used when it represented a true numerical zero. Zeros may be useful for analysis (e.g., a project with “0” stories above grade, or the impacts for a specific building element or life cycle stage are “0”). Zeros were not used as placeholders for missing information.
- **“NA” = Not applicable**. This value was used when a feature could be confirmed as not applicable to the building project (e.g., a project with only a single use type would read “NA” for secondary use type as it had none). “NAs” were not used as placeholders for missing information.
- **“NULL” = Missing information**. This was the default value for representing missing values. It indicates that information may exist for the feature, but it was not provided (e.g., a project with “NULL” for occupancy would, in reality, have building occupants, but the number of occupants was not reported). “NULL” was also used for redacted information per agreements with data contributors.

Analysis tools have different ways of dealing with blank, NULL, and NA values. For most types of analysis, users may find it easier to convert all NULLs and NAs to blank values before using the dataset files.

### Outliers and extreme data points

Five new construction building projects had total ECI values greater than 1.5x the interquartile range of all others (>~1000 kgCO_2_e/m^2^) and six had similarly high values for total MUI (>~2300 kg/m^2^). Conversely, two projects had abnormally low ECIs (<~200 kgCO_2_e/m^2^), which may be explained by their use of light-wood frame construction for modest-sized multifamily residential projects. Most notably, two of the high projects were outliers for both MUI and ECI (*project_index* numbers 26 and 160) and showed the highest ECI values of all (1638 and 2160 kgCO_2_e/m^2^, respectively). Both projects showed significantly higher MUI and ECI from steel and concrete materials compared to other projects, while displaying similar contributions of MUI and ECI from building elements and material groups. For project #26, these abnormalities may be due to its seismic design category (E) and seismic site class (D), both of which typically necessitate additional structural materials for earthquake resistance. Project #160 had the highest ECI of all and was one of only four buildings identified as a Transportation Hub in the dataset (e.g., airports, ferry terminals, bus depots, etc.). This is an understudied typology in existing ECI research and these abnormalities may be plausible for accommodating high occupancies, associated heavy infrastructure, and providing security and safety. Ultimately, while some of these projects would be considered ECI outliers using quartile-based methods, they all fall within the range of possibilities from existing research^11,12,18,19,21^, where ECIs values greater than 1000 kgCO_2_e/m^2^ or less than 200 kgCO_2_e/m^2^ are uncommon, but not unprecedented. As mentioned in our methodology, all projects were retained in the dataset unless they could be proven erroneous with the information available to us. We could not prove these data points erroneous, nor could we prove they were not. Users may prefer to filter out projects with exceedingly high or low impacts for certain use cases, or use robust summary statistics less sensitive to extreme data points. Additionally, while the dataset contains some extreme data points for ECI and MUI at the project level, these projects can still contain valuable information for analyzing building elements and materials.

## Supplementary information

Supplementary Information

## Data Availability

The code developed and used for data preparation is available in a Github repository (https://github.com/Life-Cycle-Lab/wblca-benchmark-v2-data-preparation). This code primarily leverages the Python library Pandas and Python library Pandera. The repository contains subfolders of data preparation steps for metadata processing, LCA results harmonization, and data record finalization. All of the code contains docstrings (i.e., code usage notes) to aid in interpretation and reuse.
