# Supplementary material for: A Harmonized Dataset of High-Resolution Embodied Life Cycle Assessment Results for Buildings in North America
Source: Sci Data. 2025 Jul 1;12:1085. doi: 10.1038/s41597-025-05216-0 (PMC12218139; doi:10.1038/s41597-025-05216-0)
Supplement: Supplementary file 1 — Supplementary Information [file 41597_2025_5216_MOESM1_ESM.pdf]

Supplementary Information File

Related to: “A Harmonized Dataset of High-Resolution Embodied Life Cycle Assessment Results for Buildings in North America”

Table of Contents

Additional Tables 1

Methods Section 1

Technical Validation Section 4

Additional Tables

Methods Section

Additional tables corresponding to the Methods section of the main article file are presented here.

Table S1. Mapping of the project metadata feature names used in data collection and data preparation to their corresponding final name in the data record, where applicable. This table aids in interpreting the pre-processing codes which reference feature names that were later reformatted for the final data record.

Feature Name as Collected	Feature Name in Data Record
NA	project_index
NA	site_country
Project State or Province	site_state_province
Project Climate Zone	site_clim_zone
Project Type	bldg_proj_type
Completion Year	bldg_compl_year
Building Code	bldg_code_year
Energy Code	bldg_energy_code_year
IBC Construction Type	bldg_ibc_type
Attached Parking Type	bldg_park_type
Project Floor Area	bldg_CFA
NA	bldg_GFA
Attached Parking Floor Area	bldg_parking_GFA
Added Floor Area	bldg_added_GFA
Renovated Floor Area	bldg_renovated_GFA
Primary Building Use Type	bldg_prim_use
Secondary Building Use Type	bldg_sec_use
Occupant Load	bldg_occupants
Residential Units	bldg_res_units
Stories Above Grade	bldg_stories_above
Stories Below Grade	bldg_stories_below
Building Height	bldg_height
Thermal Envelope Area	bldg_therm_env_area
Window Wall Ratio	bldg_wwr
Average R-Value Walls	bldg_rval_walls
Average R-Value Roofs	bldg_rval_roofs
Seismic Site Class	str_seis_site_cls
Seismic Design Category	str_sdc
Ultimate Wind Speed	str_wind_speed
Primary Horizontal Gravity System	str_prim_horiz_sys
Primary Vertical Gravity System	str_prim_vert_sys
Primary Lateral Force Resisting System	str_lat_sys
Podium	str_podium
Secondary Horizontal Gravity System	str_sec_horiz_sys
Secondary Vertical Gravity System	str_sec_vert_sys
Typical Column Grid, Long Direction	str_grid_long
Typical Column Grid, Short Direction	str_grid_short

Foundation Type	str_fdn_type
NA	str_sys_summary
Date of Analysis	lca_assessment_year
Design Phase	lca_design_phase
Reference Study Period	lca_RSP
Software Version	lca_software
Purpose of Assessment	lca_purp_of_assessment
NA	lca_phys_scope
Embodied Carbon Reductions Pursued	lca_ec_reductions
Embodied Carbon Percent Reduction	lca_ec_reduction_percent
Project Name	NA
LCA Report Name	NA
Date of Entry	NA
Project Description	NA
Project City	NA
Project Zip Code	NA
External Floor Area	NA
Certifications	NA
Final Report	NA
Biogenic Carbon Included	NA
Operational Energy Included	NA
Additional LCA Report Name(s)	NA
QA User Notes	NA
Building Reuse	NA
Material Reuse	NA
Alternate Structural System	NA
Structural Biobased Materials	NA
Non-structural Biobased Materials	NA
Structural Element Optimization	NA
Concrete Mix Optimization	NA
Exterior Envelope Optimization	NA
Interior Finishes Optimization	NA
Other Reductions	NA
Substructure	NA
Shell - Superstructure	NA
Shell - Exterior Enclosure	NA
Interiors - Construction	NA
Interiors - Finishes	NA
Sitework	NA
Services (MEP)	NA
Equipment & Furnishings	NA

Table S2. Project metadata validation checks using Pandera in metadata pre-processing

Collected Feature Name	Type	Check
Project State or Province<br>Project Climate Zone<br>Project Type<br>IBC Construction Type<br>Attached Parking Type	string	Matches provided dropdown values

Primary Building Use Type<br>Secondary Building Use Type<br>Seismic Site Class<br>Seismic Design Category<br>Primary Horizontal Gravity System<br>Primary Vertical Gravity System<br>Primary Lateral Force Resisting System<br>Secondary Horizontal Gravity System<br>Secondary Vertical Gravity System<br>Podium<br>Foundation Type<br>Design Phase<br>Software Version<br>Purpose of Assessment		
Project Floor Area<br>Building Height<br>Thermal Envelope Area<br>Window Wall Ratio<br>Average R-Value Walls<br>Average R-Value Roofs<br>Typical Column Grid, Long Direction<br>Typical Column Grid, Short Direction<br>Embodied Carbon Percent Reduction	float	Value > 0
Ultimate Wind Speed	float	Value > 80
Reference Study Period	int	Value = 60

Table S3: Calculated bins used for project metadata pre-processing

Field	Bins
bldg_stories_above	1<br>2 to 5<br>6 to 10<br>11 to 15<br>16 to 20<br>21 or more
bldg_stories_below	0<br>1<br>2<br>3<br>4 or more
bldg_height	0 - 7.5 m<br>7.6 - 15 m<br>15.1 - 22.5 m<br>22.6 - 30 m<br>31 - 45 m<br>46 - 60 m<br>61 - 90 m<br>Over 90 m
lca_software	Tally

	Oneclick LCA
lca_phys_scope	Sequential abbreviation listed for each Omniclass element including: (B) Substructure; (S) Shell – Superstructure; (E) Shell – Enclosure; (C) Interiors - Construction; (F) Interiors - Finishes
str_sys_summary	Concrete<br>Steel<br>Steel/Concrete<br>Steel/Masonry<br>Wood: Mass Timber<br>Wood: Light Frame<br>Wood: Hybrid

Table S4. Floor area rounding criteria used for data record finalization. All floor areas listed in the data record (CFA, GFA, Parking GFA, Renovated GFA, and Added GFA) were rounded according to these ranges.

**Range of exact floor area:**	**Rounded to the nearest:**
Less than or equal to 2,000 m^2	10 m^2
2,001 - 10,000 m^2	50 m^2
10,001 - 20,000 m^2	100 m^2
20,001 - 50,000 m^2	250 m^2
50,001 - 100,000 m^2	500 m^2
Greater than 100,000 m^2	1,000 m^2

Technical Validation Section

Additional tables corresponding to the Technical Validation section of the main article are presented here. These primarily include summary statistic values from figures within the main body.

Boxplot values were generated using Tableau Desktop which utilizes the Tukey method of quantification. This results in upper and lower "hinges" (effectively medians of the upper and lower 50% of data points) in place of pure quartiles. For large datasets like the one presented in this study, these differences are negligible. Accordingly, it presents upper and lower hinges in place of quartiles (Q1 and Q3). Notably, upper and lower hinges may vary slightly compared to pure 25th (Q1) and 75th (Q3) percentiles. All material use and impact intensity values were normalized using CFA.

Table S5. Values from Figure 3a of the main article file. Count of projects based on primary building use type.

**Primary Building Use (*bldg_prim_use*)**	**Count of Projects**	**% of Total**
Office	64	22%

Education	55	19%
Residential: Multifamily	46	16%
Public Assembly	31	11%
Laboratory	23	7.9%
Warehouse and Storage	21	7.2%
Healthcare	13	4.5%
Public Order and Safety	10	3.4%
Transportation Hub	9	3.1%
Mercantile	5	1.7%
Lodging	5	1.7%
Parking	4	1.4%
Industrial	3	1.0%
Religious Worship	1	0.3%
Food Service	1	0.3%
Other	1	0.3%

Table S6. Values from Figure 3b of the main article file. Count of projects by total floor area based on intervals as calculated by the authors.

Total Floor Area (based on calculated intervals)	Count of projects	% of Total Count
1 - 10,000 m2	148	50.68%
10,001 - 20,000 m2	61	20.89%
20,001 - 30,000 m2	35	11.99%
30,001 - 40,000 m2	16	5.48%
40,001 - 50,000 m2	7	2.40%
50,001 - 60,000 m2	6	2.05%
60,001 - 70,000 m2	6	2.05%
70,001 - 80,000 m2	2	0.68%

80,001 - 90,000 m2	5	1.71%
90,0001 m2 or more	6	2.05%

Table S7. Values from Figure 3c of the main article file. Count of projects based on LCA software used.

**Lca Software (*lca_software)***	**Count of projects**	**% of Total**
One Click LCA	93	31.85%
Tally LCA	199	68.15%

Table S8. Values from Figure 3d of the main article file. Count of projects based on stories above grade.

**Stories Above Grade (*bldg_stories_above*)**	**Count of projects**	**% of Total**
1	56	19.18%
2 to 5	146	50%
6 to 10	48	16.44%
11 to 15	21	7.19%
16 to 20	10	3.42%
21 or more	9	3.08%
NULL	2	0.68%

Table S9. Values from Figure 3e of the main article file. Count of projects based on structural system

**Primary structural system (*str_sys_summary*)**	**Count of projects**	**% of Total**
Steel	88	30.14%
Concrete	71	24.32%
Steel/Concrete	46	15.75%
Wood: Light-frame	30	10.27%
Wood: Mass Timber	30	10.27%
Steel/Masonry	12	4.11%
Other	12	4.11%

NULL	3	1.03%

Table S10. Values from Figure 4 of the main article file. Boxplot values of ECI, API, EPI, SFPI, ODPI, and NREDI for new construction projects (n=242) and life cycle stages A-C.

Impact Intensity	min	lower hinge (Q1)	median	mean	upper hinge (Q3)	max
ECI ($kgCO_2e/m^2$)	84	343	461	505	628	2160
API ($kgSO_2e/m^2$)	0.366	1.420	2.016	2.282	2.650	10.044
EPI ($kgNe/m^2$)	0.04	0.10	0.16	0.59	0.30	62.56
SFPI (kgO_3e/m^2)	3.8	20.3	26.1	29.7	34.8	272.6
ODPI ($kgCFC_{11}e/m^2$)	-1.15e-07	4.75e-06	1.17e-05	3.64e-03	2.95e-05	5.76e-01
NREDI (MJ/m^2)	756	3104	4657	5161	6594	27820

Table S11. Values from Figure 5A of the main article file. Boxplot ECI values of Omniclass elements for life cycle stages A-C of new construction project including BSECF scope (n=154). Values are listed in $kgCO_2e/m^2$.

Omniclass Element	min	lower hinge (Q1)	median	mean	upper hinge (Q3)	max
Substructure	0	25	55	75	99	669
Shell - Superstructure	20	169	257	275	349	1615
Shell - Enclosure	15	77	117	141	160	643
Interior - Construction	0	9	16	21	26	221
Interior - Finishes	4	32	52	51	67	198
Unknown	0.00	0.05	0.41	4.00	3.62	52

Table S12. Values from Figure 5B of the main article file. Boxplot ECI values of life cycle stages for new construction project including BSECF scope (n=154). Values are listed in $kgCO_2e/m^2$ and rounded to the nearest whole number.

Life Cycle Stage	min	lower hinge (Q1)	median	mean	upper hinge (Q3)	max
A1-A3	115	289	403	437	548	1929
A4	2	5	6	8	9	40
B4-B5	10	41	60	69	85	299
C2-C4	4	28	41	48	58	220

Table S13. Values from Figure 6 of the main article file. Boxplot A1-A3 MUI values for new construction projects. Values are listed in kg/m^2 and rounded to the nearest whole number.

min	lower hinge (Q1)	median	mean	upper hinge (Q3)	max
130	769	1071	1135	1388	4907

***Table S14** is on the following page.*

Table S14. Values from Figure 7 of the main article file. Boxplot A1-A3 MUI values for material groups. Values are listed in kg/m^2, sorted by descending medians, and rounded to the nearest whole number when greater than ten, to one decimal place when less than ten, or to two decimal places when less than one.

**Material Group** *(mat_group)*	**min**	**lower hinge (Q1)**	**median**	**mean**	**upper hinge (Q3)**	**max**
Concrete	0.00	564	883	915	1209	4536
Steel	3.4	41	73	87	114	573
Gypsum	0.00	8.5	29	34	54	125
Masonry	0.00	5.6	25	58	65	714
Wood and composites	0.00	0.6	5.0	31	39	316
Insulation	0.00	2.6	4.9	6.4	8.4	34
Glazing	0.00	2.1	4.4	4.8	6.7	16
Applied fireproofing	0.00	0.2	3.7	12	22	57
Cladding	0.01	0.54	2.0	4.2	5.3	30
Aluminum	0.00	0.64	1.6	3.0	3.3	27
Flooring and tile	0.00	0.44	1.5	2.4	3.4	24
Acoustic ceilings	0.00	0.25	1.2	2.1	2.2	46
Roofing and waterproofing	0.01	0.56	1.1	2.1	2.5	20
Doors and frames	0.00	0.29	0.9	1.4	1.9	26
Coatings	0.00	0.31	0.59	0.67	0.86	2.7
Windows and frames	0.00	0.13	0.53	1.6	1.7	20
Air and vapor barriers	0.00	0.11	0.39	0.75	0.99	4.6
Synthetic composites	0.00	0.08	0.21	1.0	0.71	18
Adhesives and sealants	0.00	0.05	0.15	0.19	0.29	1.1
Other metals	0.00	0.01	0.04	0.33	0.14	25
Other	0.00	0.01	0.04	0.54	0.30	14
Wall coverings	0.00	0.00	0.02	0.05	0.05	0.5
